# The Iran Thalassemia Prevention Program: Success or Failure? 

**Published:** 2015-07-20

**Authors:** M Hashemieh, H Timori Naghadeh, M Tabrizi Namini, H Neamatzadeh, M Hadipour Dehshal

**Affiliations:** 1 **Associated professor, Pediatric hematologist and oncologist, Imam Hossein Medical Center, Shahid Beheshti University of Medical Sciences, Tehran, Iran. **; 2 **Research Center of Iranian Blood Transfusion Organization, High Institute for Research and Education in Transfusion Medicine, Tehran, Iran.**; 3 **Hematology and Oncology Research Center, Shahid Sadoughi University of Medical Sciences and Health Services, Yazd, Iran.**

**Keywords:** Iran, Prevention Program, Thalassemia Belt

## Abstract

**Background:**

Iran is one of the countries located on the “thalassemia belt” and a thalassemia prevention program was approved in our country in 1995. Many different researchers have studied the success of this program with no unanimous findings.

**Material and Methods:**

A comprehensive literature search was performed using PubMed, Web of Science, and Google Scholar databases in Farsi and English languages for relevant articles published up to March 2015.

**Results:**

A total of 46 articles regarding thalassemia prevention were identified. After screening the titles and abstracts, 27 articles were excluded because they were the same articles, review articles, and case reports. Finally, 16 articles about the success of the Iranian thalassemia prevention program were selected for the evaluation.

**Conclusion:**

The findings show that the program has been significantly successful in the reduction of the new thalassemia births, though not in a few provinces like Sistan and Baluchestan. The role of the network of genetic labs has been also indispensable in the reduction of the new births. However, there is ambiguity over the impact of the program on the attitude and awareness of people across the country about the prevention of inherited diseases. However, with the success of the Iran thalassemia prevention program, it needs to be modified to be more compatible with the relevant social textures of different provinces.

## Introduction

Thalassemia major is an inherited disease most prevalent in the region called thalassemia belt (1). Iran is one of the countries located on the belt with an average thalassemia gene prevalence rate of 4% (2). Upon the establishment of Iran Thalassemia Association in 1989, the need was felt to make measures for the prevention of thalassemic newborns in Iran (2). As a result, the first country-wide thalassemia prevention program was formulated in 1995 and started to be implemented across Iran in 1997 (3). The first study about the outcomes of the program was conducted by Samavat et al. which showed significant success in decreasing the rate of thallasemic newborns (4). Subsequent studies showed the exaggerated success reported in the first study due to the delay in the registration process of newborn thalassemic patients in Iran Health System which had not been considered by the researchers (5)^. ^The second effort to evaluate the thalassemia prevention program in Iran was made by Abolghasemi et al. who considered two different phases in their study: the first phase during which the screening of marriage candidates was the only strategy to reduce the rate of thalassemia and the second phase in which the abortion in special medical cases received religious approval which made the use of prenatal diagnosis (PND (possible in prevention of thalassemia. They concluded that the former phase was not that successful and the latter phase on the contrary was success (3). However, no statistics have been reported in their study to show their definition of success. Hadipour et al. in another study evaluated the success rate of the thalassemia prevention program during 2001-2006 period indicating the downward trend in the number of thalassemia new births as a success, though with heterogeneity in a few provinces, where the program was faced with serious challenges (5). In yet another study conducted by Hadipour et al., the emphasis has been placed again on the heterogeneity of the program in different provinces, though they evaluated the program to be successful (6). Miri et al. also studied about the success rate of the program in comparison with the neighboring Muslim countries and a few European countries like Greece and Cyprus; their findings showed the program to be successful as a model for the prevention of blood-borne diseases in developing Muslim countries (2). But there is not yet any consensus on the success of the program in Iran and many other researchers like Ghotbi et al. hold the belief that the program has many obstacles in its way to fulfill its goals (7). In the present review, given the importance of thalassemia prevention in the countries being located on the thalassemia belt, and considering the importance of the Iranian prevention program as a reliable model for developing Muslim countries, we have reviewed the success of the program considering its various aspects including the change it has made in the attitude of the society, the decrease in the number of thalassemia new births, and the effectiveness of the network of PND laboratories in Iran. 

## Materials and Methods

***Search Strategy***

A comprehensive literature search was performed using PubMed and Google scholar database for relevant articles published with the following key words “thalasemia,’’ ‘‘Iran,’’ “Hemolytic disorder’’, “Beta globin deficiency’’. All eligible studies were published before March 30, 2014. In addition, studies were identified by a manual search of the reference lists of reviews and retrieved studies. We included all the case–control studies and cohort studies that investigated the thalassemia prevention program. All eligible studies were retrieved and their bibliographies were checked for other relevant publications. When the same sample was used in several publications, only the most complete study was included following careful examination.

***Inclusion criteria***

All studies were included if they met the following criteria: (1) cohort and case–control studies were considered, (2) evaluated the thalassemia prevention program. Major reasons for exclusion of studies were as follows: (1) not for thalassemia research and (2) duplicate of previous publication.

## Results

**Figure1:**
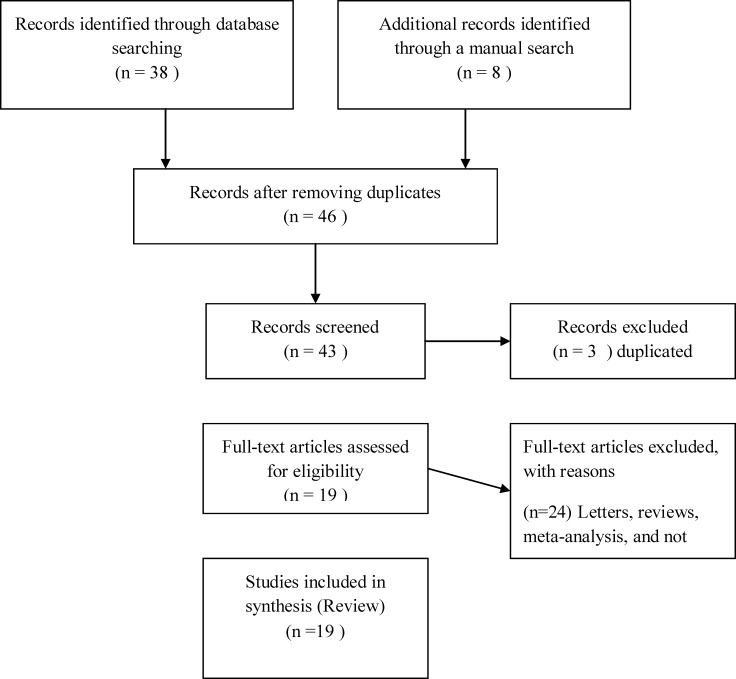
Flow chart displaying literature search and study selection

Figure 1 graphically illustrates the trial flow chart. A total of 46 articles regarding thalassemia prevention were identified. After screening the titles and abstracts, 27 articles were excluded because they were the same articles, review articles, and case reports. Finally, 19 articles had addressed the success rate of the program research-wise both country-wide and local or small-scale. Many other research efforts in this regard had been made through theses and reported in non-ISI and non-PubMed Farsi or English journals; thus, further search by Google gave us 8 more relevant articles. The published researches about the success of the program were categorized triple: those focusing on the decrease in the number of thalassemia new births, those addressing the change in the attitude of the society towards thalassemia prevention, and those evaluating the success rate of the network of PND laboratories in Iran. The findings of five of the articles were eligible to be included in more than one of the three categories above.

## Discussion

The thalassemia prevention program started in 1995 and prevailed in the whole country two years later (3). Miri et al. hold the belief that the notion of thalassemia prevention developed upon the time Iran Thalassemia Society was established^2^. The primary study on the efficacy of the program and its high success rate in shrinking the thalassemia new birth cases was conducted by Samavat and Modell in 2004; they calculated the number of the new births to be 78 in 2002 that showed 937 decreases from the estimated expectation (4). However, later studies conducted by Hadipour Dehshal et al. outnumbered the new births in 2002 to be 375 that were different from what reported by Samavat and Modell (5). The former authors have disclosed the delay in registering new thalassemia patients in the country-wide thalassemia database and it seems to be the point that the latter have been uninformed about while obtaining the false much-lower-than-expected number of thalassemia new births in 2002. The second country comprehensive study was conducted by Abolghasemi et al. (3). From the success point of view, the researchers divided the program into two phases: the first phase just based on the screening of the marriage candidates with no significant success, and the second phase in which PND was introduced with the consequent significant success. Abolghasemi et al. do not divulge any data suggesting the program success except for the same general claim herein. Their findings show that a great number of the new thalassemia birth cases post-program pertain to the couples having married before the screening to be compulsory. Nevertheless, the research findings of Hadipour Dehshal et al. show the downward trend of new thalassemia births since the very beginning years of the program and the significant increase in the efficacy of the PND lab network in Iran in 2006 (5); moreover, they hold the belief that the impact that new thalassemia births to the couples married before the implementation of the program could have on the final estimates has been dwindling gradually (5,6); thus, Hadipour et al. decline the necessity of implementing any screening plan for the couples married before 1995. Despite report differences in Samavat and Modell, Abolghasemi et al., and Hadipour Dehshal et al., all three emphasize on the program success in reducing the rate of new thalassemia births. 

Miri et al. while asserting the program success have estimated the new birth rate of thalassemia to have decreased from 864 in 1996 to 239 in 2009; this indicates of the success in prevention of the expected birth rate of 82.3% in 2009 (2). Therefore, the thalassemia prevention program is evaluated to be successful in helping the downward trend of thalassemia new births in Iran. However, Hadipour Dehshal et al. do not confirm the consistency in the success rate of the program across all the provinces of the country particularly in Sistan & Baluchestan and Kohkiluye & Boyerahmad where the efficacy of the program should be reevaluated (5,6). The former province requires special attention both for its being socially different from other provinces and its high prevalence rate of thalassemia gene. The findings of Mirimoghadam et al. are consistent with those of Hadipour Dehshal et al. about the impact of Sunni-convention unregistered marriages in the new thalassemia births thereby confirming the lack of success in this province (8). Both of the research groups refer to the high number of Baluch couples getting married based on religious traditions with the consequent delay in the registration in the official network of the country. Since premarital screening is the basis upon which the Iran thalassemia prevention program is established, the unofficially married couples would fall out of the coverage of the program (5,6,8); the offspring would comprise a great percentage of the new thalassemia births in Iran.

The research observations show the program success in the other high-rate thalassemia provinces like Fars and Mazandaran. Joulaei et al. have provided evidence for the significant decrease in the new thalassemia births in Fars province during 1997 – 2011 (9). Khorasani et al. have estimated the program success in lowering the rate of the new thalassemia cases during 1995 – 2005 in Mazandaran province (10). Ghanaei et al. claim that the rate of the new thalassemia births within the experimental implementation of the program in Isfahan province in 1993 and 1996 has fallen down to zero (11). Hadipour Dehshal et al., though not recognizing the findings of Ghanaei et al. in the eradication of the new thalassemia births in Isfahan, approve of the program success in the Iran provinces including Fars, Mazandaran, and Isfahan (5,6). The success of the program in lowering the

new thalassemia births in Isfahan was also approved by Zeinalian et al. (12) whose findings are more compatible with those of Hadipour Dehshal et al. (5,6). Haddow displays the Iran thalassemia prevention program as an appropriate model for disease prevention and control that can be used as a successful program (13). Moradi et al. acknowledge the success of the program in the screening phase but recommend some changes (14). 

The other aspects of the program including its success in the attitude and behavior change of citizens should be also taken into account. Kosarian et al. considers this aspect of the program successful in Mazandaran province (15) with which Mirimoghadam et al. disagree and further assert the necessity of awareness raising activities in Sistan & Baluchestan (8). This assertion is fully consistent with the findings of Hadipour Dehshal et al. that show the failure of the program in this province because of the inconsistencies with social norms (5,6). Miri et al. refer to how ineffective the voluntary prevention plan in Iran has been with no success in bringing about any meaningful changes in the attitude and behavior of citizens; this voluntary plan has been started to be practiced since the establishment of Iran Thalassemia Society and ended upon the implementation of the thalassemia prevention program (2). Hadipour Dehshal et al. while referring to the use of the IVF technique for thalassemia birth decline the impact of the program on the attitude and behavior change of different medical specialists particularly those not directly involved with the program (5,6). Since prenatal diagnosis (PND) plays an important role in the thalassemia prevention in Iran, Abolghasemi et al. designed a two-phase research the second of which being concerned with the use of PND in decreasing the new thalassemia births (3). Nikuei et al. approved of the success of PND in the reduction of the new thalassemia births in Hormozgan province (16). The findings of Hadipour Dehshal et al. show that the variable of "the lack of access to PND" has played a less significant role in the new thalassemia births since 2005 (5,6). They confirm the gradually ever increasing efficacy of the PND lab network in the reduction of thalassemia new births (6). Not only do Abolghasemi et al. and Hadipour Dehshal et al. emphasize on the effective role of PND in thalassemia prevention, but also on the more access of couples who are the carriers of thalassemia to PND labs (3,6). Thus, more PND efforts should be exerted to elicit more success from the program. 

## Conclusions

All studies indicate the success of the thalassemia prevention program in Iran though with no consistency in all the provinces. Therefore, the program strongly requires to be made more native and compatible with the norms of the provinces especially in Sistan & Baluchestan where we are witness to many new thalassemia cases on an annual basis. Regarding the PND lab network, all findings unanimously show the increasing efficacy in the prevention of thalassemia; however, all the researchers recommend that the decision makers be more persistent for further and easier access of the carrier couples to PND. As far as the attitude and behavior changes are concerned, the program has been of little avail in the provinces different in their social context and norms. More importantly, except for the medical practitioners directly involved in the program, other medical specialty groups should be more informed on the program and the importance of thalassemia prevention. 

Since the Iran thalassemia prevention program is represented as a distinct model among the developing and Muslim countries, the success of the program can promote the value of the thalassemia prevention in the attributed countries located on the thalassemia belt. To increase the success, it is imperative to make the program more compatible with the native parameters of the provinces and perform regular evaluations over the program. Making efforts to clarify the importance of the thalassemia prevention for the young girls and boys before the marriage age and for all medical specialties can lead to stronger public incentive for voluntary participation in thalassemia prevention. 

## Conflict of interest

The Authors have no conflict of interest.
